# The role of the tuft cell–interleukin-25 axis in the pathogenesis of inflammatory bowel disease

**DOI:** 10.3389/fimmu.2025.1629060

**Published:** 2025-09-17

**Authors:** Zishao Tao, Li Li, Ying Zhang, Yufang Tang, Simeng Zhang, Heying Yang, Guorong Jiang, Rui Zhang, Zhiwei Wu, Miao He

**Affiliations:** ^1^ School of Pharmacy, Dali University, Dali, Yunnan, China; ^2^ Department of Infectious Diseases, Nanjing Drum Tower Hospital, The Affiliated Hospital of Medical School, Nanjing University, Nanjing, Jiangsu, China; ^3^ School of Pharmacy, Shanghai Jiao Tong University, Shanghai, China

**Keywords:** tuft cell, IL-25, inflammatory bowel disease, group 2 innate lymphoid cells, microbial

## Abstract

Emerging evidence highlights the tuft cell—Interleukin-25 (IL-25) axis (tuft/IL-25 axis) as a critical orchestrator bridging luminal stimuli and intestinal immunity in Inflammatory Bowel Disease (IBD), which encompasses Crohn’s Disease (CD) and Ulcerative Colitis (UC). This review synergises current understanding of how dysregulation within this axis contributes to IBD pathogenesis, arising from disrupted immune homeostasis involving aberrant microbiota responses, genetic susceptibility, and immune pathway dysregulation. Central to this axis, intestinal tuft cells act as chemosensory epithelial sentinels, differentiating in response to microbial and metabolic cues to become the primary source of IL-25. IL-25, signaling via IL-17RB, engages innate and adaptive immune cells, particularly group 2 innate lymphoid cells (ILC2s). While IL-33-responsive homeostatic ILC2s (nILC2s) promote mucosal repair, IL-25-driven inflammatory ILC2s (iILC2s) amplify inflammation, positioning them as pivotal effectors. Critically, IL-25 exhibits a context-dependent “double-edged” role: engagement with IL-25R^+^ T cells and modulation of downstream signaling can exert anti-inflammatory effects and enhance barrier integrity, yet dysregulation drives pro-inflammatory injury. The axis is dynamically regulated by diverse luminal factors: helminth infection activates the tuft-ILC2 circuit, inducing protective type 2 immunity; specific microbial metabolites (e.g., succinate, SCFAs) modulate its activity; and viral infections can disrupt homeostasis by remodeling tuft cell function. Dysregulation of the tuft/IL-25 axis, driven by infections, microbial metabolite fluctuations, or environmental factors (including regional variations in helminth exposure linked to the hygiene hypothesis), is increasingly recognized as a significant contributor to IBD pathogenesis. Consequently, precisely regulating this axis to harness its beneficial effects while mitigating its detrimental potential represents a promising therapeutic frontier. Future strategies should integrate microbiota remodeling, targeted metabolite interventions, and potentially virus-directed therapies. Furthermore, deeper investigation into the impact of geographical environmental factors on this axis and IBD risk is warranted. Ultimately, multi-pathway approaches aimed at restoring the “immune-microbiota-epithelial” triad via reprogramming the tuft/IL-25 axis hold significant promise for novel IBD management.

## 1 Introduction

Inflammatory Bowel Disease (IBD), including Crohn’s Disease (CD) and Ulcerative Colitis (UC), is marked by persistent, recurrent inflammation of the gastrointestinal tract. The core pathology involves disrupted intestinal immune homeostasis, characterized by aberrant immune responses to commensal microbiota or dietary antigens, ultimately driving persistent inflammation and tissue damage (1, 2). Studies have established that commensal and dietary yeasts act as specific drivers of aberrant cytotoxic T helper 1 (Th1) cell responses in CD. These clonally expanded, cross-reactive CD4^+^ T cells recognize conserved fungal antigens and contribute to transmural inflammation and tissue damage through epithelial cytotoxicity (3). Under physiological conditions, intestinal homeostasis is maintained through the epithelial barrier, regulatory T cells (Treg cells), and symbiotic microbiota. In IBD, however, this equilibrium collapses due to genetic susceptibility, such as nucleotide-binding oligomerization domain-containing protein 2 (*NOD2*) mutations (4), environmental triggers (e.g., dysbiosis), and dysregulated immune pathways, including impaired Th2 regulation and hyperactivation of Th1/Th17 responses (5–7). Notably, type 2 immunity—mediated by Th2 cells and group 2 innate lymphoid cells (ILC2s)—participates in intestinal immune regulation through cytokines (IL-4, IL-5, IL-13), which promote mucus secretion and barrier function to exert protective effects (8, 9). However, excessive type 2 immune responses may also lead to the persistence and aggravation of intestinal inflammation, thereby impairing normal intestinal function (10).

As a cytokine involved in this immune balance, interleukin-25 (IL-25)—a 177-amino-acid protein—exhibits different N-terminal structural organization compared to its homologs, IL-17A and IL-17F, with longer extensions in the IL-25 protein (11). While IL-25 is synthesized by numerous immune cells, such as CD8^+^ T cells, mast cells, macrophages, dendritic cells (DCs), eosinophils, and basophils (12–14), but in the intestine, it is mainly expressed by doublecortin-like kinase 1-positive (DCLK1^+^) tuft cells (15–17). Intestinal tuft cells are relatively rare, accounting for only about 0.4% of murine intestinal epithelial cells under steady-state conditions (18, 19). These specialized chemosensory cells detect luminal signals through receptors such as bitter taste receptors (*TAS2Rs*) and, upon activation, secrete a spectrum of paracrine and endocrine mediators, including IL-25 (20, 21), acetylcholine (ACh) (22), prostaglandin D2 (PGD2) (23), thymic stromal lymphopoietin (TSLP) (24), and cysteinyl leukotrienes (CysLTs) (25). The IL-25 receptor (IL-17RB) is expressed by various immune cell types, including ILC2s (26), Th2 cells (12), DCs (27), and macrophages (28). In addition, epithelial tuft cells also express the IL-17RB (29). Among them, IL-25 derived from tuft cells can promote the effective activation of ILC2s and induce the production cytokines that participate in mediating intestinal inflammation (30). As such, IL-25 plays a key role in bridging intestinal stimulation and systemic immune homeostasis.

IBD manifests distinctly: CD can affect any segment of the gastrointestinal tract, predominantly involving the terminal ileum and colon. Its hallmark features include a “skip lesion” distribution pattern (discontinuous inflammation with intervening normal mucosa) and transmural inflammation, which may lead to complications such as fistulas, strictures, or abscess formation (31). In contrast, UC is confined to the colon and rectum and is characterized by continuous mucosal inflammation limited to the mucosa and submucosa. Typical UC lesions manifest as ulcers, crypt abscesses, and bloody diarrhea (32, 33). Current therapies targeting immune restoration—such as 5-aminosalicylic acid, immunosuppressants, and biologics—remain the cornerstone of IBD management, yet challenges such as low clinical response rates and high relapse rates persist (34). Future research should prioritize precision immunomodulation and personalized microbiota interventions to rebalance the “immune-microbiota-epithelial” triad. Within this framework, tuft cells serve as critical sentinels, orchestrating mucosal immunity by sensing lumen-derived stimuli and secreting IL-25. Their strategic positioning at the interface of luminal cues and mucosal immunity positions them as key modulators of gut infection responses and IBD pathogenesis. Given the pivotal role of the tuft cell—IL-25 axis (tuft/IL-25 axis) in initiating innate type 2 responses via ILC2s within the inflammatory milieu of IBD, this review focuses on dissecting the complex immunoregulatory crosstalk mediated by IL-25 and tuft cells in the IBD inflammatory milieu and the multifaceted pathogenesis of IBD.

## 2 Tuft cells: the central source and functional effectors of IL-25

### 2.1 Association between tuft cells and IBD

Accumulating evidence suggests a strong link between tuft cell deficiency and IBD pathogenesis. Clinical studies reveal reduced tuft cell numbers in inflamed ileal tissues of CD patients, inversely correlating with disease severity (35), and a 55% decrease in colonic tuft cells in UC patients compared to controls (36). Consistently, *DCLK1*-knockout mice (lacking tuft cells) exhibit exacerbated colitis under dextran sulfate sodium (DSS) treatment or mucin O-glycan deficiency (DKO model) (37, 38). Tuft cells inherently generate IL-25 to uphold ILC2 homeostasis in the resting lamina propria (17). Meanwhile, existing studies have emphasized the non-redundant role of ILC2s in maintaining intestinal barrier integrity and type 2 immune homeostasis. Research has demonstrated that ILC2-deficient mice fail to mount an appropriate epithelial type 2 immune response, resulting in a profound defect in worm expulsion. This deficiency is characterized by the absence of goblet cell and tuft cell hyperplasia, as well as reduced mucus production—hallmarks of an impaired type 2 immune response (39). These findings further highlight the critical role of tuft cell—ILC2 crosstalk in intestinal inflammation.

In preclinical models, IL-25 is involved in the mitigation of intestinal inflammation as a key hub in the tuft cell—ILC2s axis (40). For example, oral berberine activates bitter taste receptor signaling, promoting tuft cell differentiation and secretion IL-25, which initiates a type 2 immune response, thereby ameliorating DSS-induced colitis through ILC2 and Th2 cell modulation (41). Similarly, exogenous succinate activates succinate receptor 1 (SUCNR1) on tuft cells, inducing downstream cells to secrete IL-25 and IL-13, which drive the differentiation of intestinal stem cells (ISCs) into tuft cells and goblet cells, repairing the epithelial barrier and reducing intestinal inflammation (42, 43). However, studies have shown that succinate, whose levels are elevated in patients with IBD (44), can disrupt the immunosuppressive function of Treg cells through a FOXP3-dependent post-translational modification switch, thereby exacerbating colitis. Specifically, in Treg cells from IBD patients, succinate downregulates *Ogdh* and *Dlst* expression, reducing succinyl-CoA production, which in turn decreases succinylation of FOXP3 at lysines K8/K263. This allows STUB1-mediated ubiquitination and proteasomal degradation of FOXP3 (45). Notably, the tuft cell—ILC2 axis is further modulated by neuroimmune signaling. Neuron-derived neuromedin U (NMU) can activate ILC2s. NMU stimulation induces amphiregulin (AREG) production in ILC2s, which promotes goblet cell hyperplasia and epithelial repair, thereby sustaining type 2 immunity and epithelial homeostasis (46). Beyond the canonical IL-25—tuft cell—ILC2 axis, tuft cells also deploy an IL-25-independent effector pathway via NAIP-NLRC4 inflammasome activation and PGD2 production to engage CRTH2^+^ ILC3s, driving IL-22-mediated antimicrobial defense, as demonstrated in *Salmonella Typhimurium* infection models (47).

As the main producer of intestinal IL-25, tuft cells transmit immune signals via the IL-25-mediated tuft cell—ILC2s axis, functioning as both sentinels and effectors in mucosal immunity (48). This process involves intricate intercellular crosstalk and feedback loops.

### 2.2 Tuft cell differentiation

Lineage-tracing experiments employing an inducible Cre knock-in allele and Rosa26-lacZ reporter system in adult mice revealed that tuft cells share their origin with other epithelial cells (49, 50). Like various other intestinal epithelial cells such as enteroendocrine cells, paneth cells, and goblet cells, they originate from crypt-based columnar stem cells expressing leucine-rich repeat-containing G protein-coupled receptor 5 (*Lgr5*) (18, 51). Delta-Notch-mediated lateral inhibition controls the fate of stem cell descendants, guiding hairy enhancer of split 1 (*Hes1*)-expressing progenitors to become absorptive cells, while those expressing atonal homolog 1 (*Atoh1*) differentiate into secretory lineages like goblet cells (52, 53). Two models of tuft cell differentiation have been proposed: one suggests their derivation from non-terminally differentiated Atoh1^+^ progenitors, while the other indicates *Atoh1*-independent differentiation (54, 55). The dependency on *Atoh1* in small intestinal tuft cells is influenced by the surrounding context, with IL-13 enabling *Atoh1*-independent tuft cell generation (56). Notably, zinc-finger transcriptional repressor growth factor independent 1b (*Gfi1b*) is uniquely expressed in tuft cells but absent in other epithelial lineages, where it interacts with transcription factors *Hes1* and *Atoh1* to form a genetic network. This network functions as a Notch signaling-driven tri-stable regulatory module that regulates lineage commitment in intestinal epithelial cells (57).

Additionally, all murine tuft cells constitutively express POU class 2 homeobox 3 (POU2F3), a transcription factor critical for their development. For tuft cell development in most mucosal regions, the POU2F3 coactivator POU2AF2 is crucial. In the intestinal tract, tuft cells—apart from those in the colon and stomach—rely solely on the long isoform of POU2AF2 (58–61). Other regulators influencing intestinal tuft cell abundance include the transcription factor *SOX4* (56), the taste receptor type 1 member 3 (*TAS1R3*) (62), the cell division control protein *Cdc42* (63), and the DEAD-box RNA-binding protein DDX5 (64). Collectively, the lineage differentiation of intestinal tuft cells is intricately regulated, involving Notch signaling-mediated cell fate specification toward distinct lineages and microenvironment-dependent plasticity modulated by IL-13 and other factors.

### 2.3 Tuft/IL-25 axis signal transduction

As specialized chemosensory sentinel cells, tuft cells are activated through a diverse receptor system. Their chemosensory arsenal includes taste receptors such as *TAS1R* heterodimers for sweet and umami sensing (62), and *TAS2R* monomers for bitter compound detection (65), as well as metabolite sensors such as *SUCNR1*, which detects microbiota-derived succinate (66, 67). Additional ligand-receptor interactions include the *Shigella* metabolite N-undecanoylglycine with vomeronasal type-2 receptor 26 (*VMN2R26*) (68), and bitter substance salicin with *TAS2R143* (69), demonstrating their multimodal sensing capacities. This multimodal receptor system facilitates comprehensive luminal surveillance, integrating nutrient and microbial signals to orchestrate context-dependent immune-epithelial crosstalk. Notably, tuft cells constitutively express IL-17RB, the receptor for IL-25, independently of luminal stimuli—a critical feature of their receptor regulatory network. Through intrinsic IL-17RB signaling, these cells bind autocrine IL-25 to limit its bioavailability, thereby preventing hyperactivation of ILC2s by persistent *Il25* transcription and maintaining immune homeostasis (29).

**Figure 1:**
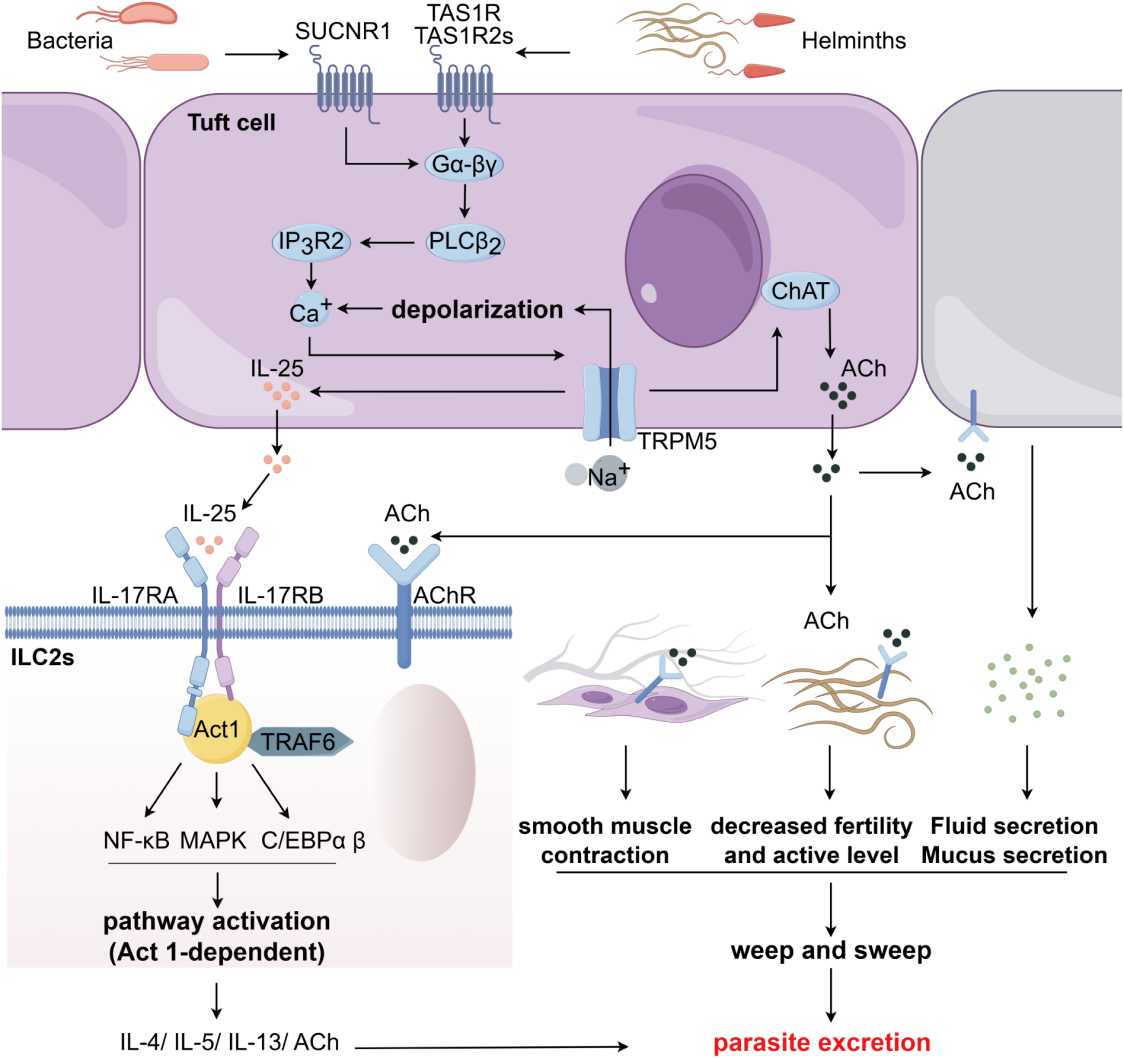
The tuft/IL-25 signaling axis. Tuft cells detect luminal pathogens (e.g., bacteria, helminths) via apical receptors, activating the *Gα-βγ*/*PLCβ2* cascade. This drives *IP_3_R2*-mediated Ca²^+^ flux, triggering membrane depolarization and IL-25 secretion. IL-25 binds IL-17RA/IL-17RB on ILC2s, activating Act1-dependent NF-κB/MAPK pathways to upregulate type 2 cytokines (IL-4/IL-5/IL-13), which promote helminth expulsion. Concurrently, tuft cell-derived acetylcholine (ACh) stimulates epithelial mucus secretion, smooth muscle contraction, and paralyzes muscarinic ACh receptor-expressing helminths, amplifying the “weep and sweep” defense to eliminate parasites.

Upon luminal stimulation, tuft cells initiate G protein-coupled signaling cascades: Gα-gustducin dissociates from Gβγ subunits, with the liberated Gβγ activating phospholipase Cβ2 (*PLCβ2*) to generate inositol trisphosphate (IP_3_). IP_3_ binding to endoplasmic reticulum IP_3_ receptor 3 (*IP_3_R3*) triggers Ca²^+^ release, while subsequent activation of the transient receptor potential cation channel subfamily M member 5 (*TRPM5*) channel mediates membrane depolarization (**Figure 1**) (70–72). These coordinated events culminate in IL-25 biosynthesis and secretion. Crucially, *IP_3_R2*-mediated Ca²^+^ mobilization has been identified as the critical step for succinate-induced IL-25 production (62, 66, 67). Secreted IL-25 engages IL-17RB receptors on ILC2s, recruiting IL-17RA to form a ternary complex (29, 73, 74). This interaction initiates *Act1* adaptor-dependent signaling, triggering *TRAF6*-mediated ubiquitination that activates NF-κB and C/EBP transcription factors. Concurrent MAPK/ERK phosphorylation cascades synergistically promote type 2 cytokine expression, orchestrating antiparasitic immunity and tissue repair (**Figure 1**) (75, 76). Such chemosensory-to-immunologic signal conversion underscores tuft cells’ pivotal role as mucosal immune regulators.

Tuft cells secrete IL-25 to activate ILC2s, which in turn produce IL-13 to promote tuft cell differentiation (20, 58). However, this IL-13-driven tuft cell hyperplasia is constrained by a bone morphogenetic protein (BMP)-dependent *SOX4* inhibition (77). BMPs belong to the TGF-β family. In addition to their traditionally recognized role in regulating bone and cartilage formation, they are also involved in regulating gastrointestinal morphogenesis, maintenance of homeostasis, ISC function, and inflammatory responses (78). Their signal transduction is achieved through the classical Smad pathway (BMP binds to type I and type II receptors, then activates SMAD1/5/8, which form a complex with SMAD4 and enter the nucleus to regulate target genes) and non-Smad pathways (such as the MAPK pathway) (79, 80). Contextually, BMP signaling maintains ISC homeostasis through spatial regulation: BMP ligands (e.g., BMP2/4) in upper crypts suppress Wnt/β-catenin signaling to limit self-renewal, while antagonists (e.g., Grem1) in crypt bases preserve the stem niche (81–83). Notably, excessive tuft cell proliferation triggers IL-13-mediated induction of BMP2/8b secretion from intestinal epithelial cells. These ligands bind BMP receptors (BMPR1A/BMPR2) on ISCs, activating SMAD1/5/8 phosphorylation and nuclear translocation. The phospho-SMAD complex then recruits HDAC co-repressors to the *SOX4* promoter, epigenetically silencing its transcription (77, 78). This pathway suppresses the development of SOX4^+^ tuft cell progenitors, establishing a self-limiting negative feedback loop (77). These findings highlight IL-25’s pivotal regulatory role in intestinal immune homeostasis: it not only initiates epithelial-immune crosstalk as an “alarmin”, but also orchestrates cellular lineage equilibrium through bidirectional regulatory circuits.

## 3 IL-25R^+^ ILC2s: the pivotal effectors and regulators within the tuft/IL-25 axis

### 3.1 Intestinal ILC2s: context-dependent functions

Intestinal ILC2s exhibit unique phenotypic and functional properties within the tuft cell—ILC2 axis. Notably, ILC2s can exert protective functions in specific contexts. In the DSS-induced murine acute colitis model, administration of exogenous recombinant murine IL-33 (rmIL-33) significantly ameliorated intestinal inflammation. Treg cells and ILC2s function as primary cellular targets of IL-33 to mediate this tissue-protective effect (84). Consistently, studies demonstrate that upon NMU stimulation, ILC2s produce AREG, which potentiates intestinal barrier repair during DSS-induced colitis, thereby effectively mitigating intestinal inflammation (46). Furthermore, they can produce IL-13 to recruit myeloid-derived suppressor cells (MDSCs), thereby reducing pro-inflammatory Th1/Th17 cells and improving intestinal barrier integrity (85). However, the pathological conversion of ILC2s can drive inflammatory diseases and tumor progression. In colorectal cancer (CRC), the IL-25—ILC2—MDSC axis suppresses anti-tumor immunity and fosters the formation of an immunosuppressive tumor microenvironment (86). Conversely, some studies have also demonstrated that in CRC, ILC2s are associated with enhanced tumor protection, strengthened anti-tumor immunity, and reduced metastatic dissemination. Indeed, the pro-tumor versus anti-tumor roles of ILC2s in CRC remain controversial (87, 88). An inulin fiber-enriched diet has been shown to induce iILC2s (ILC2^INFLAM^) expressing *Tph1* in the mouse colon; these cells produce IL-5 but fail to produce the tissue-protective factor AREG, leading to eosinophil accumulation and exacerbation DSS-induced intestinal damage (89). Concurrently, the ILC2^INFLAM^ produces type 2 inflammatory cytokines IL-5 and IL-13, which recruit eosinophils and exert dual immunomodulatory effects: conferring protection against helminth infections while promoting the pathogenesis of allergic diseases (90).

### 3.2 Plasticity and heterogeneity of IL-25-responsive ILC2s

Regarding ILC2 subtypes, IL-33-responsive ILC2s residing in pulmonary and adipose-associated lymphoid tissues have been designated as homeostatic or natural ILC2s (nILC2s), while naming the KLRG1^hi^ cells that emerge exclusively upon IL-25 stimulation or infection are referred to inflammatory ILC2s (iILC2s) (91). Studies also show that IL-25 elicits iILC2s, which exhibit functional plasticity (iILC2s can differentiate into transient progenitor cells of nILC2-like cells), IL-17A production, high expression of the activation marker KLRG1, and low CD90/*Thy1* expression (91, 92). Other studies have further defined two distinct ILC2 subsets based on surface marker expression: iILC2s, characterized as Lin^-^ CD45^+^ CD127^+^ ST2^-^ KLRG1^hi^, and nILC2s, identified as Lin^-^ CD45^+^ CD127^+^ ST2^+^ KLRG1^int^ (93). Notch signaling drives this functional plasticity of iILC2s by inducing *Rorc* transcription, thereby mediating the conversion of nILC2s into iILC2s (94). Furthermore, studies demonstrate that iILC2s co-express high levels of GATA-3 alongside low amounts of RoRγt, enabling the coexistence of key functional attributes from both ILC2 and ILC3 subsets. This cell responds to IL-25 signaling and possess the capacity for dual IL-13/IL-17 production (91, 94). Similarly, iILC2s can be reprogrammed into IL-17-producing ILC3-like cells upon exposure to specific cytokines or during fungal infections. Over time after IL-25 exposure, a subset of lung iILC2s acquires ST2 expression while losing IL-25 receptor expression, phenotypically resembling nILC2s (95).

ST2^-^ KLRG1^hi^ iILC2s are characterized by high *Il17rb* expression and selective responsiveness to IL-25, in contrast to nILC2s, which predominantly express the IL-33 receptor (*Il1rl1*/ST2) and respond to IL-33 (91, 96). iILC2s serve as early producers of IL-13 and IL-4 during helminth infection (day 5 post-infection), preceding cytokine secretion by nILC2s, and can migrate from gut to lung to initiate anti-helminth immunity (96). Studies have revealed that lung-resident iILC2s, following colonization with the murine intestinal protozoan symbiont *Tritrichomonas musculis*, resemble IL-25-induced intestinal ILC2s. These iILC2s display lower levels of CD90, CD127, and ST2 (*Il1rl1*), but higher levels of inducible ICOS, *Il17rb*, RoRγt, OX40L, *Il5*, *Il13* and *Il17a* (97). Furthermore, Adoptive transfer of total leukocytes isolated from CD45.1^+^ *Rag1* ^−/−^ deficient mice into CD45.2^+^ *Rag1* ^−/−^ deficient recipients, followed by IL-25 administration, revealed that only KLRG1^+^ ILC2s from the small intestinal lamina propria (siLP) robustly expanded into iILC2s in recipient lungs. In contrast, bone marrow-derived cells yielded minimal iILC2s, and lung-derived cells generated none, demonstrating that peripheral IL-25-induced iILC2s originate from intestinal-resident ILC2 subsets (98).

### 3.3 Activation mechanisms of intestinal iILC2s

RNA-seq analysis revealed a remarkable transcriptomic similarity between intestinal ILC2s and IL-25-induced iILC2s, providing higher-resolution and conclusive evidence for their common intestinal origin (98). However, the mechanisms underlying their activation in the gut require further investigation. Intraluminal succinate injection into small intestinal organoids (SIOs) significantly upregulated the tuft cell marker *DCLK1* and increased *Il17e* transcription. When co-cultured with succinate-treated SIOs, ILC2 precursors (ILC2Ps) differentiated into a higher proportion of KLRG1^+^ ILC2s, demonstrating that metabolite-activated tuft cells drive ILC2 maturation and expansion via IL-25 secretion (99). The relative expression levels of ILC2-related genes in the small intestine were determined in *Vil1* ^Cre^ *; Il17rb* ^fl/fl^ mice (which lack IL-17RB specifically in intestinal epithelial cells, resulting in tuft cell deficiency of IL-17RB) and *Il17rb* ^fl/fl^ mice (with normal IL-17RB expression) (29). The results revealed that the ILC2 signature genes, including *Gata3*, *Rora*, *Id2*, *Il5*, and *Thy1*, exhibited comparable expression levels between the two groups. This consistent expression ensures the preservation of their canonical ILC2 identity. In contrast, genes such as *Klrg1*, *Icos*, and *Nmur1*, along with ILC2 activation-associated genes (e.g., *Il10*, *Il13*, *Cd69*, *Tigit*, *Il10rb*, and *Cxcr6* among others), were significantly upregulated in *Vil1* ^Cre^ *; Il17rb* ^fl/fl^ mice. These dynamically upregulated genes collectively show the core molecular signature of the iILC2 phenotype: iILC2s are characterized by high expression of KLRG1 and low expression of CD90 (96), along with elevated levels of IL-13, ICOS, and TIGIT (97, 98). Furthermore, when the expression of surface markers on colonic ILC2s in *Rag2* ^−/−^ mice infected with *Strongyloides ratti* (following treatment with a pan-retinoic acid receptor inverse agonist, RAi) shifts toward a non-intestinal phenotype (such as *Il-17rb* ^lo^ CD90^hi^ KLRG1^lo^ ICOS^hi^), it impairs the responsiveness of ILC2s to immune signals like IL-25 and disrupts mucosal defense mechanisms critical for anti-helminth immunity (100). Some studies suggest that iILC2s are absent from peripheral tissues under steady-state conditions but can be induced at multiple sites by helminth infection or IL-25 administration (101). We postulate that the synergistic interplay of these mediators, with IL-25 serving as the primary initiator, drives the differentiation of intestinal ILC2s into iILC2s. Among these regulatory mechanisms, the expression of IL-17RB in tuft cells is pivotal for maintaining ILC2 homeostasis—its deficiency results in the sustained stimulation of intestinal ILC2s by IL-25, thereby promoting their transition toward an inflammatory phenotype. However, the activation of intestinal ILC2s serves merely as a necessary condition for the generation of iILC2s; their full differentiation requires additional regulatory factors. The precise relationship between these two cell populations remains to be fully elucidated.

Collectively, ILC2s function as pivotal effectors within the tuft/IL-25 axis, exhibiting remarkable functional plasticity that dictates IBD outcomes. Their dichotomous role—switching between tissue-repairing (IL-33/nILC2-driven) and inflammation-promoting (IL-25/iILC2-driven) states—directly influences mucosal homeostasis and immune homeostasis.

## 4 IL-25 signaling in IL-25R^+^ T cells: balancing protective and pro-inflammatory responses in IBD

### 4.1 Anti-inflammatory effects and homeostasis maintenance

IL-25 exerts protective roles in colitis by suppressing intestinal inflammation and maintaining immune homeostasis, primarily through modulating Th2 responses and inhibiting Th1/Th17 pathways. In DSS-induced acute colitis, recombinant IL-25 (rIL-25) elevates colonic IL-23 and TGF-β1, thereby alleviating intestinal inflammation and tissue damage. Within this IL-25-primed milieu, heightened IL-23 may cooperate with TGF-β1—which is produced via IL-25/IL-13 signaling and enhances IL-23R expression—to establish an anti-inflammatory network that suppresses Th1 responses and promotes repair. The mechanistic details of this interaction nevertheless warrant further validation (102). IL-25 is also proposed as a critical anti-inflammatory cytokine in trinitrobenzene sulfonic acid (TNBS)-induced colitis, where miR-31-mediated targeting of IL-25 suppresses IL-12/23-dependent Th1/Th17 inflammatory responses (103). Commensal bacteria-induced IL-25, secreted by intestinal epithelial cells (IECs), negatively regulates Th17 cell expansion by suppressing the IL-23/IL-17 pathway, thereby maintaining gut immune homeostasis (104). Additionally, IL-25 reduces the production of TNF-α, IFN-γ, and IL-17A in IBD CD4^+^ T cells while promoting IL-10 secretion, thereby inhibiting Th1/Th17 differentiation (105). In colorectal cancer models, IL-25 blockade increases tumor burden (106). All of this underscores its role in suppressing pro-inflammatory microenvironments. Critically, IL-25’s ability to maintain intestinal homeostasis aligns with, and may be supported by, functional autophagy—a fundamental cellular process essential for gut equilibrium. Autophagy supports intestinal equilibrium by clearing intracellular pathogens and damaged components, preserving epithelial barrier integrity, and regulating immune responses—processes essential for creating the stable cellular environment in which IL-25’s anti-inflammatory effects (e.g., Th1/Th17 suppression, IL-10 promotion) can optimally function (107, 108). Consequently, autophagy impairment, a recognized contributor to IBD pathogenesis, likely undermines the mucosal conditions necessary for IL-25’s full protective potential.

### 4.2 Pro-inflammatory effects and pathological aggravation

Conversely, IL-25 may exacerbate inflammation by amplifying Th2-mediated immune responses. Notably, UC exhibits a predominance of Th2-related cytokines (e.g., IL-5, IL-13) (109), rendering this pro-inflammatory effect particularly relevant to its pathogenesis. In oxazolone-induced UC, blocking IL-25 signaling with IL-25 neutralizing antibodies significantly improves colitis, implicating the role of IL-25 in Th2-driven pathology (110). Administering IL-25 systemically to naïve mice triggers the expression of IL-4, IL-5, and IL-13, driving Th2 polarization alongside elevated serum IgE, eosinophil infiltration, and mucosal abnormalities (e.g., mucus hypersecretion and epithelial hyperplasia) (111). IL-25 also upregulates colonic epithelial IL-33, IL-6, and TNF-α, forming a pro-inflammatory feedback loop that exacerbates tissue damage (112). Epithelial-specific IL-25 deletion reduces IL-1β, IL-6, TNF-α, and CCL2 levels in DSS-treated mice, correlating with decreased colitis-associated tumorigenesis (113).

In Th1/Th17-dominant inflammation, IL-25 suppresses hyperactive innate immunity and pro-inflammatory pathways, exerting protective effects. However, under Th2-skewed conditions, its overexpression disrupts immune equilibrium, driving pathological cascades. Thus, therapeutic strategies targeting IL-25 must consider the inflammatory context to achieve precise intervention, balancing its “double-edged sword” effects to restore mucosal integrity and immune homeostasis. The paradoxical dual effects of IL-25 in IBD—anti-inflammatory protection versus pro-inflammatory injury—are intriguing and the currently focus of the research.

## 5 Microbial and metabolic regulation of the tuft/IL-25 axis

The human gut microbiota, a diverse community comprising viruses, fungi, and bacteria, plays essential roles in food digestion and microenvironment maintenance. Dysbiosis—characterized by disrupted symbiosis between commensal and pathogenic microbes and reduced microbial diversity—is a key factor in IBD pathogenesis (114). For instance, IBD patients exhibit decreased relative abundances of *Firmicutes* (e.g., *Lachnospiraceae*) and *Bacteroidetes*, alongside increased *Proteobacteria* (115). In UC, *Eubacterium rectale* and *Akkermansia muciniphila* are depleted, while *Escherichia coli* is enriched (116). Comparative analyses have demonstrated marked elevations of *Actinomyces*, *Eggerthella*, *Clostridium III*, *Faecalicoccus*, and *Streptococcus* in CD/UC cohorts versus healthy controls, in contrast to significant depletions of *Gemmiger*, *Lachnospira*, and *Sporobacter* (117). Notably, the invasive strain *Fusobacterium nucleatum*, isolated from CD patients, is implicated in CD progression and colorectal carcinogenesis (118). This all suggests that such dysbiosis disrupts intestinal barrier integrity, triggering aberrant immune activation and inflammation. Emerging research continues to unravel the multifaceted crosstalk among IBD, gut microbiota dysbiosis, host metabolic reprogramming, and immune dysregulation (119–121). Recent studies highlight the therapeutic potential of medicinal fungi such as *Hericium erinaceus*, whose polysaccharides and alcohol extracts demonstrate anti-inflammatory effects in IBD models by modulating gut microbiota composition (e.g., increasing *Lachnospiraceae* and *Eubacterium*) and suppressing pro-inflammatory cytokines (TNF-α/NF-κB) while enhancing IL-10 production (122–124). Notably, intestinal microbiota and their metabolites coordinate intestinal immune homeostasis by modulating the tuft/IL-25 axis, which is crucial for the regulation of the inflammatory environment in IBD (35, 125, 126). Global epidemiological patterns reveal an inverse relationship between the prevalence of IBD and helminth infections—a cornerstone of the “hygiene hypothesis.” This hypothesis posits that reduced exposure to symbiotic microorganisms and parasites in early life dysregulates immune development, increasing susceptibility to immune-mediated disorders such as IBD (127). This section explores how the gut microbiome and its metabolites regulate the intestinal tuft/IL-25 axis, a critical pathway implicated in IBD pathogenesis and potentially underlying the protective effects suggested by the hygiene hypothesis.

### 5.1 Helminths trigger tuft-ILC2 circuitry for type 2 immunity and barrier repair

Notable human-infecting species include *Ascaris lumbricoides*, *Trichuris trichiura*, *Necator americanus*, and *Ancylostoma duodenale* (128, 129). Intestinal tuft cells act as epithelial sentinels that promote type 2 immunity against helminths (130). Infection of mice with parasites such as *Heligmosomoides polygyrus*, *Trichinella* sp*iralis*, or *Nippostrongylus brasiliensis* universally increases tuft cell abundance, indicating a conserved response to helminth colonization (20). Tuft cells secrete IL-25 and CysLTs to activate ILC2s by recognizing worms such as *Nippostrongylus brasiliensis*, *Heligmosomoides polygyrus*, and *Trichinella* sp*iralis*, initiating a feedback loop to promote tuft cell proliferation (25). Studies indicate that tuft cells initiate IL-5/IL-13 signaling by emitting the alarmin cytokine IL-25, which in turn activates ILC2s. Once activated, ILC2s produce type 2 cytokines like IL-4, IL-5, and IL-13 (131). Especially, studies have identified a novel colonic IL-4^+^ ILC2 subpopulation that constitutively expresses IL-4 independently of the classical alarmin pathway (unaffected by IL-25 injection or IL-33/TSLP deletion), with its development of this subset is regulated through the vitamin B1 (VB1)-dependent metabolic axis involving glucose-6-phosphate dehydrogenase (G6PD) and the pentose phosphate pathway (PPP), establishing a unique stimulatory mechanism distinct from microbiota-derived signals and conventional cytokine networks (132). These cytokines signal via IL-13Rα1/IL-4Rα heterodimeric receptors on ISCs, driving their differentiation into tuft and goblet cell lineages. This feed-forward loop amplifies the immune response, promotes epithelial remodeling, and ultimately facilitates parasite expulsion (58). Tuft-goblet cell expansion restores the damaged epithelial barrier through enhanced tight junctions and mucus layer formation, creating a physical shield between immune cells and luminal microbiota (133). Notably, IL-13 secreted by ILC2s in the lung initiates an autoregulatory feedback circuit through bidirectional ILC2—T cell crosstalk, amplifying IL-2-dependent ILC2 proliferation and Th2 cell polarization, which further increases type 2 cytokine production and tissue remodeling. We speculate that a similar circuit may operate in the intestine (17, 134, 135).

Additionally, tuft cells, which uniquely express choline acetyltransferase (ChAT) within the intestinal epithelium (48), orchestrate antiparasitic defense through other signaling axes. Helminth-activated tuft cells release IL-25, driving ILC2-dependent IL-13 production that stimulates tuft cell hyperplasia and subsequent ACh release into the lumen. ACh exerts tripartite effects: stimulating neighboring epithelial cells to secrete fluid, binding to muscarinic ACh receptors on smooth muscle to enhance contractility (136, 137), and reducing pathogen fecundity via parasite-expressed muscarinic receptors (138). This “weep and sweep” mechanism not only facilitates parasite expulsion but also ameliorates gut dysbiosis in IBD by restoring microbial homeostasis, ultimately attenuating intestinal inflammation (**Figure 1**). Notably, ILC2s activated by tuft-derived IL-25 are also key ACh producers, wherein autocrine muscarinic signaling enhances their proliferation and type 2 immunity to bolster anti-parasite defense (139). In allergy, ILC2-derived ACh shows dual effects: promoting eosinophilia via autocrine loops while inhibiting neutrophil recruitment through *CXCL1/2* suppression and macrophage modulation (140). Thus, the ChAT/ACh pathway also operates as an effector arm of the core tuft/IL-25 axis.

### 5.2 Microbial metabolites activate the tuft/IL-25 axis to modulate IBD inflammation

The gut microbiota is acknowledged as a crucial factor in the development of IBD. Microbially derived metabolites serve as critical molecular mediators of interactions between the gut microbiome and host immunity or metabolism. For example, the fermentation of dietary fiber by gut microbiota results in the production of short-chain fatty acids (SCFAs), which are key metabolites that exhibit anti-inflammatory properties and regulate intestinal immune responses and barrier integrity (141). *Clostridium* sp*orogenes*, a human commensal bacterium, generates metabolites such as indole-3-propionic acid (IPA), branched-chain fatty acids (BCFAs) such as isobutyrate and isovalerate, and SCFAs (142, 143). These metabolites orchestrate crosstalk among gut bacteria, IECs, antigen-presenting cells, and lymphocytes. Intestinal tuft cells sense SCFAs and BCFAs through G protein-coupled receptor 41 (Gpr41, encoded by *FFAR3*) receptor signaling, triggering their activation and expansion to promote epithelial regeneration, these metabolites also inhibit intestinal inflammation via enhanced IL-22 production and Foxp3^+^ Treg cells differentiation (144). During helminth infection, cytosolic phospholipase A2 (cPLA2) in tuft cells liberates arachidonic acid from phospholipids, which is subsequently metabolized by enzymes (e.g., Pla2g4a, Alox5ap, Ltc4s) into leukotrienes that synergize with IL-25 to activate ILC2s through CysLT receptors, thereby amplifying type 2 immune responses (25). These metabolites activate ILC2s to secrete IL-13 through different pathways, driving tuft and goblet cell proliferation and mucus hypersecretion, thereby facilitating the “weep and sweep” mechanism for parasite clearance (17). This potential metabolic communication network could facilitate tuft cell proliferation in response to luminal microbiome perturbations, which would be a mechanism potentially critical for suppressing intestinal inflammation and restoring epithelial barrier integrity. Moreover, this axis may play a key role in coordinating tuft/IL-25-mediated improvement in IBD inflammation.

### 5.3 Virus-mediated tuft cell functional remodeling and immune homeostasis

The impact of viruses on the tuft cell compartment adds another layer of complexity to host-microbe interactions relevant to IBD. IBD patients may develop immunocompromised status due to pathological manifestations or immunosuppressive therapies (e.g., corticosteroids, anti-TNF-α agents), resulting in heightened susceptibility to viral infections (e.g., *Epstein-Barr virus* (EBV), *cytomegalovirus* (CMV), and *enteroviruses*) (145, 146). Tuft cells, rare chemosensory epithelial cells, serve dual roles as viral targets and immune mediators. *Murine norovirus* (MNVCR6) exploits *CD300lf*, an immunoregulatory receptor uniquely expressed on tuft cells, to establish persistent infection within this immune-privileged niche. Remarkably, the introduction of MNVCR6 into germ-free mice repairs the intestinal barrier, adjusts immune cell populations, and promotes homeostasis (147–150). Similarly, *enteroviruses* (e.g., EV71, CVA16, CVB3/4) enhance IL-25 expression and tuft cell expansion via folate metabolism. Folate metabolism, essential for purine and methyl donor synthesis, supports IL-25-mediated tuft cell expansion during RNA viral infections (151). Furthermore, EV71 induces functional remodeling of intestinal tuft cells by stimulating IL-25 production. This not only drives tuft cell expansion but also confers immune memory, enabling a potent secondary response characterized by enhanced IL-25 secretion and SAT1-mediated polyamine depletion to restrict viral replication (152). *Rotavirus* (RV) infection induces functional specialization within the intestinal tuft cell compartment: infected mature tuft cells activate antiviral defense pathways, while simultaneously inducing neighboring immature tuft cells to enhance luminal surveillance. This coordinated remodeling, which couples pathogen sensing in mature cells with heightened environmental monitoring in precursor cells, demonstrates a sophisticated mechanism for maintaining immune homeostasis during viral challenge (153, 154).

## 6 Conclusion

**Figure 2:**
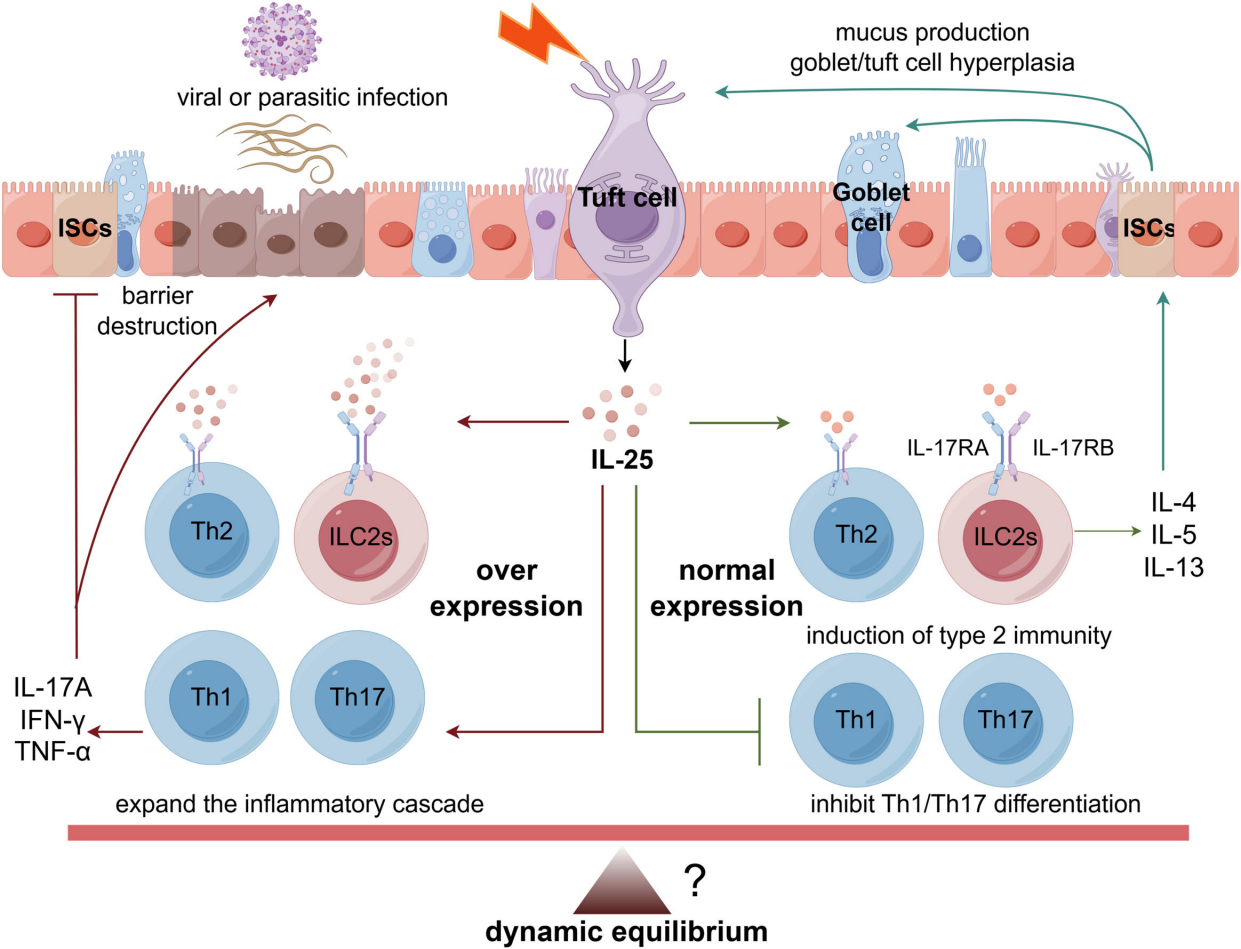
The tuft/IL-25 axis modulates intestinal immune homeostasis by enhancing Th2-mediated barrier restoration and suppressing Th1/Th17-driven inflammation. Under viruses or parasites infection, imbalance in this dynamic equilibrium leads to hyperactivation of Th1/Th17 immunity or amplification of Th2 inflammatory cascades, resulting in epithelial damage, barrier dysfunction, and progression of IBD.

Epidemiological evidence reveals a significant inverse correlation between helminth exposure and IBD prevalence, with developing regions exhibiting high helminth infection rates demonstrating substantially lower IBD incidence compared to low-exposure developed nations (127, 146). Critically, the tuft/IL-25 axis plays an important role in these geographical IBD disparities, with tuft cells acting as luminal sentinels that sense diverse stimuli (helminths, microbial metabolites, viruses) and secrete IL-25 to orchestrate mucosal immunity. This signaling activates ILC2s and modulates the Th1/Th17-Th2 balance—a process exhibiting fundamental context-dependent duality: suppressing inflammation in Th1/Th17-dominant milieus while potentially exacerbating pathology in Th2-skewed environments. However, persistent axis activation—whether through chronic helminth infection, unresolved viral persistence, or other triggers—disrupts immune homeostasis, initiating a detrimental cycle of “proinflammatory-repair dysregulation.” This aberrant signaling promotes inflammatory dissemination and accelerates irreversible tissue damage (including fibrosis) through dysregulated metabolic-immune crosstalk (155–157) (**Figure 2**). Ultimately, dysregulation driven by dysbiosis, viral persistence, helminth deficiency (hygiene hypothesis), or metabolite fluctuations disrupts the essential “immune-microbiota-epithelial” triad, fueling IBD pathogenesis.

Future therapeutic paradigms must therefore prioritize precision modulation of IL-25/ILC2 signaling. Critically, geographical disparities in helminth exposure modulate IBD risk through the tuft/IL-25 axis. Defining dynamic equilibrium thresholds for helminth/viral exposure across diverse regions may enable precise therapeutic targeting of IL-25/ILC2 signaling. Such strategies should be integrated with microbiota remodeling, metabolite intervention, and viral-host equilibrium management to concurrently mitigate immune exhaustion, control persistent viral reservoirs, and restore intestinal barrier integrity. This multimodal approach could reprogram epithelial-immune crosstalk, potentially offering novel pathways toward sustained remission in IBD.
